# Next-Generation Sequencing of *DDX41* in Myeloid Neoplasms Leads to Increased Detection of Germline Alterations

**DOI:** 10.3389/fonc.2020.582213

**Published:** 2021-01-28

**Authors:** Sarah A. Bannon, Mark J. Routbort, Guillermo Montalban-Bravo, Rohtesh S. Mehta, Fatima Zahra Jelloul, Koichi Takahashi, Naval Daver, Betul Oran, Naveen Pemmaraju, Gautam Borthakur, Kiran Naqvi, Ghayas Issa, Koji Sasaki, Yesid Alvarado, Tapan M. Kadia, Marina Konopleva, Rashmi Kanagal Shamanna, Joseph D. Khoury, Farhad Ravandi, Richard Champlin, Hagop M. Kantarjian, Kapil Bhalla, Guillermo Garcia-Manero, Keyur P. Patel, Courtney D. DiNardo

**Affiliations:** ^1^ Department of Clinical Cancer Genetics, The University of Texas M. D. Anderson Cancer Center, Houston, TX, United States; ^2^ Department of Hematopathology, The University of Texas M. D. Anderson Cancer Center, Houston, TX, United States; ^3^ Department of Leukemia, The University of Texas M. D. Anderson Cancer Center, Houston, TX, United States; ^4^ Department of Stem Cell Transplantation, The University of Texas M. D. Anderson Cancer Center, Houston, TX, United States

**Keywords:** germline, hereditary, MDS, AML, DDX41

## Abstract

Previously considered rare, inherited hematologic malignancies are increasingly identified. Germline mutations in the RNA helicase *DDX41* predispose to increased lifetime risks of myeloid neoplasms with disease often occurring later in life which presents challenges for germline recognition. To improve identification of germline *DDX41*, individuals presenting with ≥1 *DDX41* alteration on an institutional MDS/AML next-generation sequencing based panel with at least one at >40% variant allele frequency were flagged for review and genetic counseling referral. Of 5,801 individuals, 90 (1.5%) had ≥1 *DDX41* mutation(s) identified. Thirty-eight (42%) patients with a median age of 66 years were referred for genetic counseling; thirty-one were male (81.5%). Thirty-five (92%) referred patients elected to pursue germline evaluation and in 33/35 (94%) a germline *DDX41* variant was confirmed. Twenty-two patients (66%) with germline variants reported antecedent cytopenias, seven (21%) had a prior history of malignancy, and twenty-seven (82%) reported a family history of cancer. Predictive genetic testing for healthy family members under consideration as stem cell transplant donors was successfully performed in 11 family members, taking an average of 15 days. Near-heterozygous *DDX41* mutations identified on next-generation sequencing, particularly nonsense/frameshift variants or those at recurrent germline “hot spots” are highly suggestive of a germline mutation. Next-generation sequencing screening is a feasible tool to screen unselected myeloid neoplasms for germline *DDX41* mutations, enabling timely and appropriate care.

## Introduction

Over the past decade, identification of and clinical testing for inherited predispositions to hematologic malignancies has emerged as one of the fastest growing areas of cancer genetics. These germline predispositions were historically thought to be rare; however, recent studies have identified mutations in 11–37% of individuals with hematologic malignancies referred for genetic evaluation (1–4). Growing awareness of germline predisposition syndromes has raised questions about how to best identify, test, and manage individuals who carry germline mutations (5, 6). In 2016 the World Health Organization included germline predisposition mutations in their revision of the classification of myeloid neoplasms (2).

Germline mutations in the gene *DDX41* were identified as a susceptibility to myeloid neoplasms in 2015 (7–9). The incidence of germline *versus* somatic etiology of identified *DDX41* mutations in MDS/AML patients remains poorly defined. *DDX41* mutations have been described in approximately 1–3% of MDS/AML patients and are also described to a lesser extent in multiple myeloma, lymphoma, chronic myelomonocytic leukemia (CMML), and myeloproliferative neoplasms (MPNs). Unlike many other predisposition genes, the penetrance of *DDX41* mutations is predicted to be relatively modest, although exact penetrance data are not available. Furthermore, the average age of MDS/AML onset in mutation carriers is notably older at 65 years, than in many cancer predisposition syndromes and overlaps with the general population of sporadic MDS/AML diagnoses (7, 9, 10). This low penetrance often results in a limited or absent family history, which prevents identifying patients who might otherwise be flagged for genetic evaluation based on family history criteria. The later age of diagnosis also precludes identification by age-based criteria for early-onset disease. Therefore, individuals with *DDX41* mutations may go unrecognized if held to “standard” family history and/or age-based criteria for genetic evaluation (5). Optimal clinical care of these patients, however, does rely on identifying these underlying mutations; *DDX41*-related donor-derived leukemia has been described in several individuals with MDS/AML who underwent hematopoietic stem cell transplantation (HSCT) from matched related donors (MRD) found later to carry the familial *DDX41* mutation (11, 12). Furthermore, lenalidomide has been suggested as an effective treatment strategy for myeloid malignancies with *DDX41* mutations [and without del(5q)] based on case reports and retrospective analyses (13, 14).

Individuals with hematologic malignancies routinely undergo somatic molecular analysis of the bone marrow by next-generation sequencing (NGS) for diagnosis, prognostication, and treatment selection. With the availability and use of NGS-based panels for such analysis, it is increasingly apparent that variants, which appear to be somatically acquired, may instead be germline (inherited) (15–17). We hypothesized that indirect assessment of the *DDX41* gene *via* an MDS/AML NGS prognostication panel may increase detection of germline mutation carriers. Herein we describe the identification and characterization of patients with *DDX41* germline mutations identified through the incorporation of *DDX41* mutation testing in patients with hematologic malignancies.

## Materials and Methods

The *DDX41* gene has been analyzed since April 2017 as part of the standard myeloid NGS mutation panel at The University of Texas M. D. Anderson Cancer Center. From April 2017 to December 2019, 5,801 patients who were evaluated for possible hematologic malignancy underwent molecular analysis by NGS. Diagnoses of individuals undergoing NGS testing included acute lymphoblastic leukemia (ALL), AML, biphenotypic leukemia, chronic lymphocytic leukemia (CLL), chronic myeloid leukemia (CML), hairy cell leukemia (HCL), MDS, MDS/MPN, and MPN. Ninety individuals (1.5%) were identified to have one or more *DDX41* mutations at >40% variant allele frequency (VAF) in the bone marrow. Ordering providers were notified to consider genetic counseling referral for these patients based on the near-heterozygous allelic frequency suggesting possible germline origin. Patients were referred for genetic evaluation at the treating physician’s discretion. Clinical data including medical history and pathology data were obtained from the electronic medical record; family history data were obtained by a genetic counselor.

### Genetic Evaluation

Standard genetic counseling evaluation by a board-certified genetic counselor was conducted for all referred individuals. Consenting individuals then underwent site-specific testing of the identified somatic *DDX41* mutation, full *DDX41* gene sequencing, and/or multigene panel testing of MDS/AML predisposition genes including *DDX41*, as indicated based on personal and/or family history. Genetic testing in all cases was performed by a Clinical Laboratory Improvement Amendments (CLIA)-approved commercial genetic testing laboratory on DNA extracted from cultured skin fibroblasts obtained from a four millimeter skin punch biopsy.

## Results

### Demographics and Germline Testing Outcomes

Thirty-eight out of 90 (42%) individuals with *DDX41-*mutated hematologic neoplasms at near-heterozygous frequency were referred for genetic counseling and offered germline testing. Demographic and clinicopathologic data are summarized in **Table 1** and **Supplemental Table 1**. The majority were male (81.5%), and the average age at diagnosis of myeloid neoplasm was 66 years (range: 48–85 years). Regarding race and ethnicity, thirty five (92%) self-identified as non-Hispanic White, two (5%) as Hispanic White, and one (3%) as Asian. Presenting myeloid malignancies were varied including 18 (47%) with MDS, 15 (39%) with AML, three (7%) with therapy-related myeloid neoplasm (t-MN), one (3%) with chronic lymphocytic leukemia (CLL), and one (3%) referred to our institution for cytopenias with concern for MDS without confirmation of hematologic malignancy.

**Table 1:** Characteristics of germline DDX41 mutation carriers (n = 33).

Characteristic	Patients, n (%)
**Sex**	
Male	26 (79)
Female	7 (21)
**Age at hematologic malignancy, years**	
Median	67
Range	54–85
**Family history of hematologic malignancy and/or cytopenias**	13 (39)
**Personal history of prior malignancy**	8 (24)
**Personal history of cytopenias**	22 (66)
**Hematologic Malignancy**	
MDS-MLD	6 (18)
MDS-RAEB	8 (24)
MDS/MPN	1 (3)
AML	13 (39)
t-MN	3 (9)
CLL	1 (3)
None	1 (3)
**Cytogenetics**	
Diploid	26 (79)
Trisomy 8	2 (6)
-Y	3 (9)
der(1;14)	1 (3)
Not available	1 (3)
**Somatic Mutations**	
≥1 somatic *DDX41* mutation	21 (64)
Other genes	18 (55)

MDS-MLD, myelodysplastic syndrome with multilineage dysplasia, MDS-RAEB, myelodysplastic syndrome-refractory anemia with excess blasts, MDS/MPN, myelodysplastic syndrome/myeloproliferative neoplasm, AML, acute myeloid leukemia, t-MN, therapy-related myeloid neoplasm, CLL, chronic lymphocytic leukemia.

**Figure 1:**
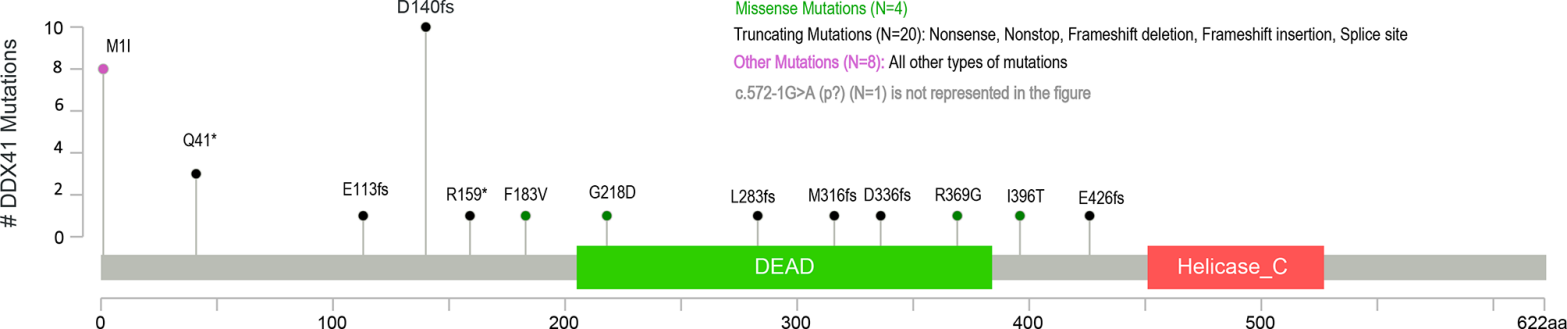
Identified germline *DDX41* variants.

All patients had at least one *DDX41* mutation identified by NGS panel which met the threshold for germline evaluation. Thirteen patients harbored a single *DDX41* variant; 64% were in previously described “hot-spot” germline *DDX41* mutations, p.D140fs*2, p.M1I, or p.Q41*. Intronic variants (c.434+1G>A) or missense mutations (p.Gly218Asp, p.Met155Ile, p.I396T) were identified in the remaining cases (**Figure 1**). The majority of patients (n = 25, 71%) had two (biallelic) or more *DDX41* mutations. The recurrent somatic mutation c.1574G >A (p.R525H) was the most common secondary mutation in individuals with ≥2 variants, present in over half of patients. One patient had three *DDX41* alterations: p.M1I, p.R525H, and p.A346T.

Thirty-five of the 38 patients referred for genetic counseling elected to undergo germline *DDX41* genetic testing *via* skin fibroblast analysis and thirty-three (94%) patients were confirmed to have a germline *DDX41* variant. Twenty-eight of the 33 variants identified as germline were nonsense/frameshift or splice site mutations and classified as pathogenic or likely pathogenic variants (**Figure 1**). Three individuals were found to carry missense mutations (p.F183V, p.G218D, p.R369G) which were classified as variants of uncertain significance. No patient harbored more than one germline *DDX41* variant. Two individuals with missense *DDX41* variants with VAF ≥40% (p.M155I in one patient, and one patient with both p.Y259C and p.R525H) on somatic sequencing tested negative for germline *DDX41* variant(s). These two patients comprised the youngest diagnoses in this series: MDS at age 53 and AML at age 48.

Three individuals did not undergo germline testing: one deferred testing during the initial consultation and has not rescheduled, and two declined testing (including only one patient declining due to concern about the skin biopsy procedure).

### Disease Characteristics, Treatment, and Survival

The majority of patients with confirmed germline *DDX41* mutations had normal (diploid) cytogenetics (79%); three had −Y, two had trisomy 8, one had der (1;14), and one was unknown. The most common somatic mutation was a second *DDX41* mutation, which occurred in 71% of patients with confirmed germline mutations. Concomitant *ASXL1* mutations were seen in six patients (18%), and no other somatic mutations occurred with notable frequency. Additional somatic mutations and their variant allele frequencies are detailed in **Supplemental Table 1**.

Most confirmed *DDX41* germline patients were treated with hypomethylating agents or other low-intensity therapies (61%), nine with intensive chemotherapy (27%), one MDS patient was treated with lenalidomide, and three underwent observation with supportive care. At the time of this analysis, 26 (79%) patients with germline variants are alive with a median overall survival of 37.4 months.

Of the 35 patients evaluated for germline *DDX41* status, 11 (31%) underwent allogeneic hematopoietic stem cell transplantation (HSCT). Eight received transplants from related donors, including four matched sibling donors (MRD) and four haploidentical donors. In five cases, the related donor tested negative for the familial *DDX41* mutation prior to transplant. In two cases, the *DDX41* status of the donor was unknown. In one case, the related donor was positive for the *DDX41* mutation; however, the mutation was not identified in the family until after transplant. The average time from first consultation at our institution to HSCT was 360 days, or 11.8 months (range 33–2,729 days). Pre-HSCT genetic testing for the familial *DDX41* mutation in potential related donors took an average of 14.8 days (range 8–22 days). In the patients who underwent matched related or haploidentical HSCT, a total of 11 potential related donors underwent predictive genetic testing, with an average of 2.5 relatives tested for each patient in the pre-transplant setting (range 1–5).

### Personal and Family History

The majority of patients (66%) with germline *DDX41* variants had a known prior history of antecedent cytopenias: twelve with leukopenia, four with anemia, and six had bicytopenias (most often leukopenia and anemia). Seven (21%) had a prior history of malignancy; including three (9%) with a previous history of hematologic malignancy (one each with non-Hodgkin lymphoma (NHL), chronic lymphocytic leukemia (CLL), and polycythemia vera (PV)). Additionally, one of the patients who declined germline confirmation of a *DDX41* p.M1I mutation had prior history of monoclonal gammopathy of uncertain significance (MGUS). Six patients (18%) had a prior solid tumor diagnosis, three of which were prostate cancer. The patient with NHL also had a previous history of melanoma. Three patients had received prior therapy with chemotherapy and/or radiation therapy (NHL, ovarian cancer, CLL). Additionally, two patients (6%) had a reported history of autoimmune disorders (rheumatoid arthritis in a patient harboring p.IVS6-1G>A, Grave’s disease in an individual harboring p.Leu283Cysfs*21).

Regarding family history, 77% of patients evaluated for potential germline *DDX41* mutation reported a family history of cancer and/or hematologic disorder. Fifteen (43%) had a family history of hematologic malignancies (AML, lymphoma, MDS, aplastic anemia, biphenotypic leukemia, leukemia not otherwise specified) and/or cytopenias (leukopenia, thrombocytopenia). Nine individuals reported a family history of both solid tumors and hematologic malignancies.

### Genetic Testing in Unaffected Relatives

Thirteen unaffected relatives underwent predictive genetic testing, including eleven unaffected relatives who were under consideration as potential HSCT donors. Two individuals desired to undergo predictive genetic testing outside of the context of transplant planning. Of the 13 unaffected relatives who underwent genetic testing, five tested positive for the familial mutation (two males) and eight tested negative (three males). Unaffected mutation carriers were offered biannual surveillance and follow up in the hereditary hematologic malignancy clinic (HHMC).

## Discussion

The relatively low penetrance and older age at onset of *DDX41*-related malignancy presents significant challenges to the identification of individuals harboring germline mutations. To evaluate whether an NGS-based molecular panel designed primarily for somatic mutation assessment could be utilized to improve upon identification, individuals harboring one or more somatic variants in *DDX41* at near-heterozygous VAF (defined as >40%) were considered for referral for germline evaluation.

Thirty-eight individuals with myeloid malignancies were referred for and elected to undergo genetic counseling. The average age of diagnosis of our cohort was 66 years, consistent with previous reports and validating the long latency of malignancy in these patients (9, 13, 18, 19). Of interest, the majority of individuals found to harbor germline variants were male, similar to previous reports demonstrating a male predominance in individuals with *DDX41* mutated neoplasms (7, 9, 18, 19). Since the individuals with germline mutations in this cohort were ascertained from somatic sequencing, which is performed on all patients with myeloid malignancies regardless of sex, sex selection bias is likely mitigated. *DDX41* is located on autosomal chromosome 5q35 which precludes sex-linked inheritance. This could represent increased penetrance of MDS/AML development in male *DDX41* germline mutation carriers, however the biological mechanism of this phenomenon is unexplored and, given the higher proportion of hematologic malignancy in white males as well as their population majority, additional population and family studies are needed.

The vast majority of patients elected to proceed with germline testing, when offered, and were confirmed to carry one of the *DDX41* variants identified first by somatic NGS analysis in the germline. Recurrently reported “hot spot” germline mutations at *DDX41* p.M1I, p.D140fs, and p.Q41* represented two thirds of our cohort and were notably always present in the germline when seen on somatic panel testing. To our knowledge, no individuals analyzed were related; however, the high percentage of non-Hispanic White race/ethnicity in this cohort is consistent with the increased prevalence of European founder mutations. One patient in our series had biallelic *DDX41* mutations on NGS, c.1105C>G (p.R369G) and c.11C>G (p.S4*). Surprisingly, germline analysis identified the p.R369G variant as germline (classified as a variant of unknown significance) and the p.S4* nonsense mutation was not present in the germline. Quesada et al. reported this particular patient in a cohort of probable, but unconfirmed, somatic and germline *DDX41* mutations; germline testing performed in this case confirmed the missense p.R369G variant as germline and the frameshift mutation as somatic (19).

Early reports indicated germline *DDX41* mutations are associated with high grade neoplasms and diploid karyotype with relatively poor outcomes, although more recent reports show more encouraging trends in outcomes (9, 18). In our cohort, germline mutation carriers presented primarily with high-risk MDS and AML with MDS-related changes. Consistent with previous reports, the majority of MDS/AMLs harbored diploid cytogenetics. While our cohort presented with predominantly high grade neoplasms, treatment and disease outcomes were relatively better than expected based on previous reports.

Several studies have demonstrated antecedent cytopenias, particularly leukopenia, in germline *DDX41* mutation carriers (7). Polprasert and colleagues described red cell macrocytosis and monocytosis in germline carriers (9), while Sebert et al. described cytopenias in about half of their cohort; however, the affected lineage was not specified (18). In our cohort, two-thirds of mutation carriers had a history of antecedent cytopenia, the majority of which were leukopenia, sometimes presenting years to decades prior to hematologic malignancy diagnosis. With the continued validation of antecedent leukopenia in our cohort and others, it may be prudent to consider germline testing in individuals with idiopathic leukopenia, particularly if there is a known family history of hematologic malignancy.

Three male germline carriers had a prior history of prostate cancer. All were treated with either surgery or hormone therapy likely indicating low grade tumors. The occurrence of prostate cancer in this cohort is most likely a reflection of the older, predominantly male population, although future studies are warranted to evaluate this potential association. The majority of patients reported a family history of cancer. Like previous studies, reported hematologic malignancies were primarily myeloid (AML, MDS) but also included lymphoid malignancies (Hodgkin and NHL), biphenotypic leukemia, aplastic anemia, and peripheral cytopenias (leukopenia, thrombocytopenia) (7).

All unaffected relatives of patients with pathogenic germline variants were offered genetic testing in the context of genetic counseling. Pre-test counseling for potential HSCT donors included education regarding the genetics, natural history, and risks associated with germline *DDX41* mutations, inheritance pattern, *a priori* risk, implications for their affected relative, and implications for their own health based on the genetic test results. Predictive testing of potential HSCT related donors was frequently performed during the HSCT planning phase, with results obtained in an average of 15 days. Thus, we confirm that timely genetic testing can identify optimal family member donors without leading to significant delays in the time to HSCT.

While 90 individuals were initially flagged with *DDX41* somatic mutations at or near heterozygous VAF, only 38 were referred for genetic counseling and evaluation. Limitations to this study include a small patient population, dependence on physician referral, patient motivation to complete germline genetic testing, and short median follow-up time. Additionally, lymphoid malignancies were not screened for somatic *DDX41* mutations and therefore potential germline carriers with lymphoma and/or multiple myeloma may have been missed. However, the data presented here confirm and validate the previously reported characteristics of germline *DDX41* mutation carriers, better define antecedent cytopenias, and further support the inclusion of *DDX41* on myeloid NGS panels for the purpose of identifying germline mutation carriers.

In conclusion, the detection of somatic *DDX41* mutations at near-heterozygous frequencies on NGS panel testing, particularly when a recurrent “hot spot” germline mutation or nonsense/frameshift variant is present, is highly suggestive of a germline mutation. NGS panel testing, through multidisciplinary collaboration, provides a feasible mechanism to screen unselected hematologic malignancy patients for high likelihood of germline *DDX41* mutations. The early identification of germline mutation carriers enables timely and appropriate confirmation of germline mutation status, screening of potential related and/or haploidentical HSCT donors, and an opportunity to improve patient outcomes.

## Data Availability Statement

The original contributions presented in the study are included in the article/**Supplementary Material**. Further inquiries can be directed to the corresponding author.

## Ethics Statement

The studies involving human participants were reviewed and approved by The University of Texas MD Anderson Institutional Review Board (IRB). The patients/participants provided their written informed consent to participate in this study.

## Author Contributions

SB, CD, MR, and KP contributed to the conceptualization of the project. SB collected and analyzed data and wrote and revised the manuscript. FJ contributed figures and reviewed the manuscript draft. MR, GM-B, KT, ND, BO, NP, GB, KN, GI, KS, YA, TK, MK, RS, JK, FK, RC, HK, KB, and GG-M contributed data for analysis, reviewed and edited drafts of the manuscript. All authors contributed to the article and approved the submitted version.

## Conflict of Interest

The authors declare that the research was conducted in the absence of any commercial or financial relationships that could be construed as a potential conflict of interest.

The reviewer CH declared a past co-authorship with one of the authors CD to the handling editor.
