# Supplementary material for: Next-Generation Sequencing of DDX41 in Myeloid Neoplasms Leads to Increased Detection of Germline Alterations
Source: Front Oncol. 2021 Jan 28;10:582213. doi: 10.3389/fonc.2020.582213 (PMC7878971; doi:10.3389/fonc.2020.582213)
Supplement: Supplementary file 1 [file Table_1.docx]

**Supplementary Table 1. Demographic and clinicopathologic characteristics of individuals undergoing germline *DDX41* testing (n=35)**

| **Pt #** | **Sex** | | **Age at dx (years)** | **Disease** | **Germline *DDX41* mutation** | **ACMG Variant Interpretation** | **Somatic *DDX41* mutation(s) [VAF %]** | **Cytogenetics** | **Co-Occurring Somatic Mutation(s) [VAF %]** | **Therapy** | **Prior cytopenias** | **Prior malignancy** | **OS**  **(months)** | **Family History** | **Vital Status** |
| --- | --- | --- | --- | --- | --- | --- | --- | --- | --- | --- | --- | --- | --- | --- | --- |
| 1 | M | 67 | | AML | c.3A>G (p.M1I) | Pathogenic | none | normal | none | HMA, MRD-SCT | Leukopenia, thrombocytopenia | None | 38.4 | AML (brother), lymphoma (mother), thrombocytopenia (son) | A |
| 2 | F | 60 | | AML | c.572-1G>A | Pathogenic | c.1574G>A (p.R525H) [4.7] | 46,XX,der(1;14) | *JAK2* [8.8]*,* | Intensive therapy, haplo-SCT | Leukopenia | None | 8.4 | Breast (mother) | D |
| 3 | M | 58 | | MDS, AML | c.3G>A (p.M1I) | Pathogenic | c.1574G>A (p.R525H) [15.0], c.1036G>A (p.A346T) [9.6] | normal | none | Intensive therapy | Anemia | None | 28.0 | Lung (mother) | D |
| 4 | M | 57 | | AML | c.415_418dupGATG (p.D140fs*2) | Pathogenic | none | normal | none | Intensive therapy, MRD-SCT | None | None | 19.1 | none | A |
| 5 | M | 77 | | MDS-MLD | c.3G>A (p.M1I) | Pathogenic | none | normal | *DNMT3A* [5.9] | HMA-based | None | Prostate | 45.9 | Prostate, pancreas (paternal grandfather) | A |
| 6 | F | 62 | | RCMD | c.121C>T (p.Q41*) | Pathogenic | c.962CT> (p.P321L) [28.7] | normal | none | Observation | Leukopenia | None | 45.5 | none | A |
| 7 | M | 68 | | AML | c.415_418dupGATG (p.D140fs*2) | Pathogenic | c.1574G>A (p.R525H) [1.8] | normal | none | HMA-based | None | None | 8.3 | Lung (sister), prostate (brother), colon (brother), breast (niece) | A |
| 8 | M | 70 | | MDS-EB | c.415_418dupGATG (p.D140fs*2) | Pathogenic | c.694A>G (p.T232A) [3.6] | normal | none | HMA-based, MUD-SCT | Leukopenia | None | 7.4 | Prostate (father, paternal uncle) | A |
| 9 | F | 60 | | t-MDS | c.547T>G (p.F183V) | VUS | c.682G>T (p.G228C) [8.2] | normal | *ASXL2* [1.3] | HMA-based, haplo-SCT | None | Melanoma, NHL | 6.9 | MDS (father), leukemia NOS (paternal aunt), MDS (paternal uncles x2) | A |
| 10 | M | 71 | | AML | c.1273_1276dupCTCG (p.E426fs) | Pathogenic | c.1574G>A (p.R525H) [22.9] | normal | *ASXL1* [29.1], *ASXL1* [20.9], *PHF6* [35.9], *STAG2* [5.5], *U2AF1* [2.0] | HMA-based | Leukopenia | Prostate | 6.0 | Leukemia NOS (paternal aunt), pancreas (father) | A |
| 11 | M | 53 | | MDS-EB | n/a |  | c.776A>G (p.Y259C) [8.0], c.1574G>A (p.R525H) [9.5] | 46,XY,inv(9) | *CBL* [10.8]*, EZH2* [1.9]*, EZH2* [2.4], *SUZ12* [4.8], | HMA-based, MRD-SCT | None | None | 13.5 | Thrombocytopenia (sister) | A |
| 12 | M | 72 | | RAEB | c.415_418dupGATG (p.D140fs*2) | Pathogenic | c.1574G>A (p.R525H) [1.1] | normal | *TET2* [3.3]*, SH2B3 (c.703C>T)** [62.9] | HMA-based | None | Prostate | 7.0 | Leukemia NOS (father, paternal aunt), Hodgkin’s lymphoma (paternal aunt) | A |
| 13 | M | 74 | | AML | c.121C>T (p.Q41*) | Pathogenic | none | normal | *SRSF2* [6.9]*, BCORL1* [7.3]*,* | HMA-based | None | None | 5.1 | Colon (father), breast (mother, maternal aunt) | A |
| 14 | M | 85 | | RAEB | c.3G>A (p.M1I) | Pathogenic | c.1574G>A (p.R525H) [1.4] | 45,X,-Y | None | HMA-based | Leukopenia | None | 48 | Breast (daughter), cervical (daughter) | A |
| 15 | M | 80 | | MDS-EB | c.946_947del (p.M316fs) | Pathogenic | c.1574G>A (p.R525H) [5.4] | normal | None | HMA-based | Leukopenia | None | 12 | MDS (sister), breast (paternal aunt, paternal grandmother) | A |
| 16 | M | 80 | | CLL | c.3G>A (p.M1I) | Pathogenic | none | normal | None | Observation | Anemia, thrombocytopenia | None | 24 | Unknown (adopted) | A |
| 17 | M | 74 | | MDS-EB | c.434+1G>A (IVS5+1G>A) | Pathogenic | none | 47,XY,+8 | None | HMA-based | Leukopenia, anemia | None | 12 | Colorectal (mother) | A |
| 18 | F | 74 | | AML | c.3G>A (p.M1I) | Pathogenic | none | normal | *ASXL1* [3.8]*, BCOR* [33.7], *GNAS* [6.3]*, PHF6* [7.1] | HMA-based | Anemia | None | 12 | Unknown | A |
| 19 | M | 73 | | MDS/MPN | c.475C>T (p.R159*) | Pathogenic | none | normal | *JAK2* [2.4] | HMA-based | Leukopenia | PV | 60 | Prostate (father), breast (sister) | A |
| 20 | M | 73 | | t-AML | c.415_418dupGATG (p.D140fs*2) | Pathogenic | c.1574G>A (p.R525H) [2.1] | normal | None | HMA-based | None | CLL |  | Colon (mother, father) | A |
| 21 | M | 71 | | AML | c.3G>A (p.M1I) | Pathogenic | none | normal | *ASXL1* [5.8]*, CUX1* [4.3], *TET2* [6.7] | Low-intensity | None | Melanoma | 12 | AML (father), MDS/AML (paternal aunt) | A |
| 22 | M | 71 | | MDS/AML | c.337del (p.E113fs) | Pathogenic | c.1574G>A (p.R525H) [6.5] | 45,X,-Y | *EED* [3.2]*, TET2* [1.3] | HMA-based | Leukopenia | None | 6 | Cytopenias (father), breast (mother, sister) | A |
| 23 | M | 67 | | AML | c.415_418dupGATG (p.D140fs*2) | Pathogenic | none | normal | *NF1*[2.8] | HMA-based, MUD-SCT | Leukopenia | None | 60 | Leukemia NOS (mother), neutropenia (brother), brain cancer (maternal aunt) | A |
| 24 | M | 65 | | MDS | c.415_418dupGATG (p.D140fs*2) | Pathogenic | c.962C>T (p.P321L) [22.1] | normal | None | HMA-based | Leukopenia | None | 96 | none | A |
| 25 | M | 65 | | MDS-EB/AML | c.653G>A (p.G218D) | VUS | none | normal | None | Intensive therapy | Anemia | None | 12 | none | A |
| 26 | F | 65 | | t-MDS | c.1187T>C (p.I396T) | Pathogenic | c.1756G>A (p.G586W) [27.7] | 47,XX,+8 | *ASXL1* [13.4]*, TP53* [1.4]*, PHF6* [24.9] | HMA-based, MRD-SCT | None | Ovarian | 12 | Lung (mother) | A |
| 27 | M | 62 | | MDS-EB | c.415_418dupGATG (p.D140fs*2) | Pathogenic | c.1574G>A (p.R525H) [4.6] | 45,X,-Y | None | Lenalidomide | Leukopenia, anemia | None | 24 | none | A |
| 28 | M | 61 | | AML | c.3G>A (p.M1I) | Pathogenic | none | normal | *SETBP1* [2.7] | Intensive therapy | None | None |  | Multiple myeloma (mother), Leukemia NOS (paternal aunt), AML (paternal cousin, paternal grandfather) | D |
| 29 | F | 60 | | None | c.415_418dupGATG (p.D140fs*2) | Pathogenic | none | normal | None | Observation | Macrocytic anemia | None | n/a | AML (maternal aunt), Leukemia NOS (maternal grandmother) | A |
| 30 | M | 60 | | AML | c.121C>T (p.Q41*) | Pathogenic | c.1589G>A (p.G530D) [8.7] | normal | None | Intensive therapy | Leukopenia, thrombocytopenia | None | 11 | Breast (paternal grandmother, paternal cousin) | A |
| 31 | M | 57 | | AML | c.415_418dupGATG (p.D140fs*2) | Pathogenic | c.1574G>A (p.R525H) [2.4] | unknown | None | Intensive therapy, MRD-SCT | None | None | 120 | Thrombocytopenia (mother), colon (paternal grandmother) | A |
| 32 | M | 57 | | MDS-MLD | c.1105C>G (p.R369G) | VUS | c.11C>G (p.S4*) [5.8] | normal | *ASXL1* [7.8]*, TP53* [2.5] | HMA-based | Leukopenia | None |  | MDS (mother) | A |
| 33 | M | 55 | | MDS-MLD | c.1004dupT (p.D336fs) | Pathogenic | c.962C>T (p.P321L) [7.3] | normal | *ASXL1* [19.8]*, NRAS* [11.5]*, SRSF2* [2.2] | HMA-based, haplo-SCT | Leukopenia, anemia | None | 24 | Neutropenia (father) | A |
| 34 | F | 54 | | MDS-EB | c.847del (p.L283fs) | Pathogenic | none | normal | None | Intensive therapy | Leukopenia | None | 7 | None | A |
| 35 | M | 48 | | AML | n/a |  | c.465G>A (p.M155I) [1.8] | complex | None | Intensive therapy, MUD-SCT | None | None |  | AML (maternal uncle) | D |

M: male; F: female; AML: acute myeloid leukemia; MDS: myelodysplastic syndrome, not otherwise specified; MDS-MLD: myelodysplastic syndrome with multilineage dysplasia; RCMD: refractory cytopenia with multilineage dysplasia; MDS-EB: myelodysplastic syndrome with excess blasts; RAEB: refractory anemia with excess blasts; MGUS: monoclonal gammopathy of uncertain significance; t-MDS: therapy-related myelodysplastic syndrome; PV: polycythemia vera; CLL: chronic lymphocytic leukemia; VUS: variant of uncertain significance; VAF: variant allele frequency; HMA: hypomethylating agent; MRD-SCT: matched related donor stem cell transplant; MUD-SCT: matched unrelated donor stem cell transplant; OS: overall survival; A: alive; D: deceased.
*variant was unable to confirmed on germline sample, denoted for high VAF (>60%)
